# Supplementary material for: In vitro reconstitution of herpes simplex virus 1 fusion identifies low pH as a fusion co-trigger
Source: mBio. 2023 Oct 24;14(6):e02087-23. doi: 10.1128/mbio.02087-23 (PMC10746285; doi:10.1128/mbio.02087-23)
Supplement: Supplemental Figures — Figures S1–S9. [file mbio.02087-23-s0001.pdf]

Supplementary Information

In-vitro reconstitution of Herpes Simplex Virus 1 fusion identifies low pH as a fusion co-trigger

J. Martin Ramirez, Ariana Calderon-Zavala, Ariane Balaram, and Ekaterina E. Heldwein

Supplementary Figures S1-S9

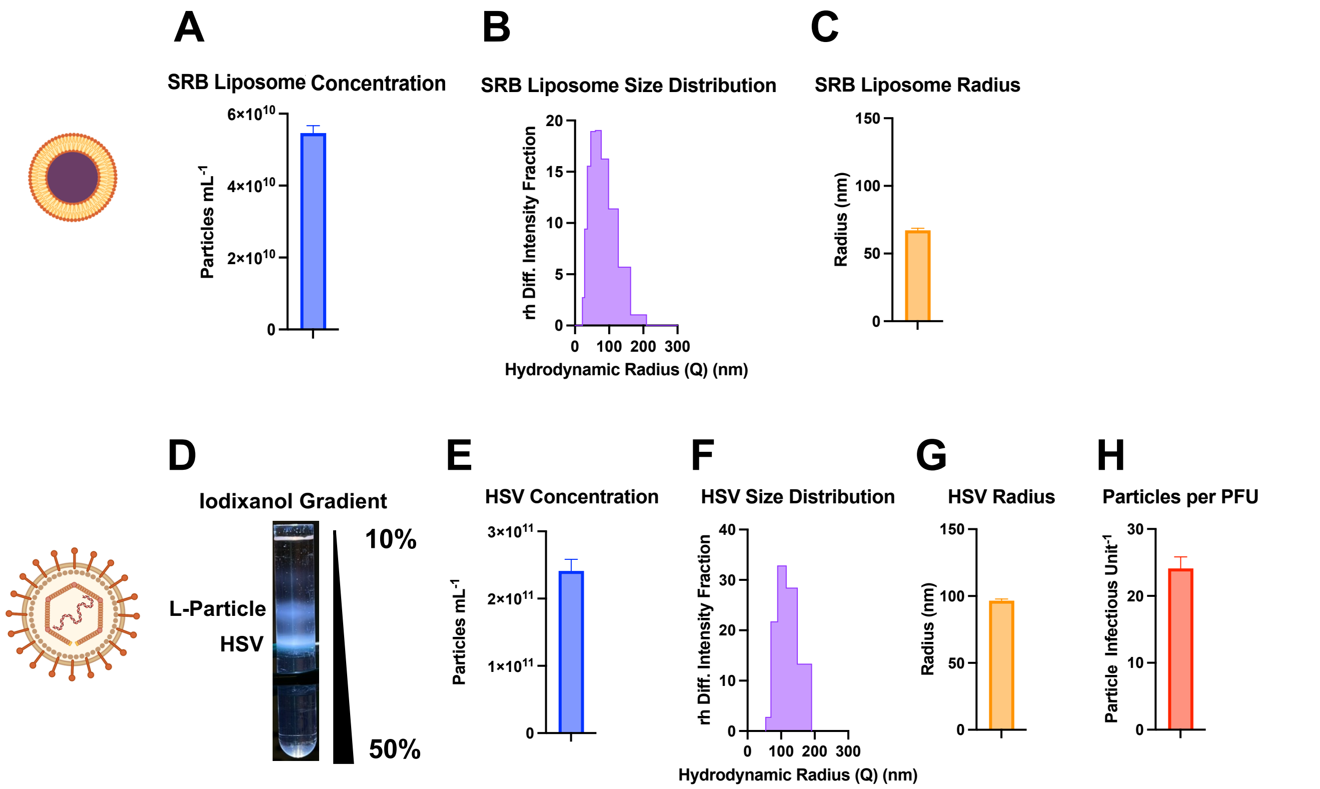

Supplementary Figure S1. Physical measurements and preparation of reagents required for bulk fusion optimization. (**A-C)** Representative static and dynamic light scattering measurements of liposomes including particle concentration (**A**), size distribution (regularization) (**B**), and radius of gyration (**C**). (**D)** HSV-1 density centrifugation purification using iodixanol to separate intact HSV-1 virions from light particles (L-particle). (**E-H)** Representative static and dynamic light scattering measurements of HSV-1 including particle concentration (**E**), size distribution (**F**), a radius of gyration (**G**), and particles per PFU (**G**). Bar height is the mean static light scattering (concentration and radii) value obtained in the same time interval used to determine the size distribution from dynamic light scattering. Error bars represent the SEM. All light scattering data were obtained using a Dawn Heleos II with DLS and processed with the software Astra 7.3.

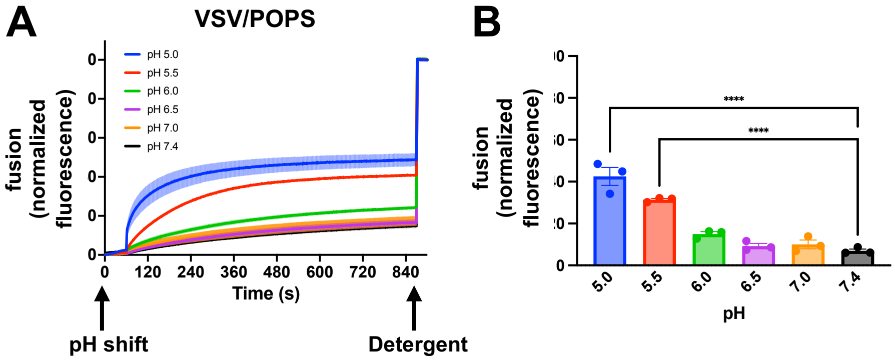

Supplementary Figure S2. SRB VSV Fusion has similar pH value as previously reported in the literature, validating the SRB fusion assay. VSV was incubated with SRB-containing POPS LUVs at different pH using the workflow described in Fig 1C. Increase in SRB fluorescence due to its dilution and dequenching reported on fusion. Lipid composition is listed in Table 1. Fluorescence was recorded and normalized to fully dequenched fluorescence by using the equation: $F_{normalized}=F_t-F_i / (F_{max}-F_i)^{-1} \ x \ 100\%$, where F_t is fluorescence at time *t,* F_i is initial fluorescence and F_{max} is fluorescence after the addition of Triton X-100. **(A)** For each condition, representative traces of single biological replicates, each consisting of three technical replicates, are shown. Curves represent the mean values, and the shaded area, the SEM. **(B)** Extent of fusion at 5 min post-triggering (acidification). Each data point is a biological replicate representing a mean normalized fluorescence from 3 technical replicates. Bars represent the mean values, and the error bars, the SEM. Each condition was done in triplicate. *: $p < 0.05$, **: $p < 0.01$, ***: $p<0.001$ ****: $p<0.0001$.

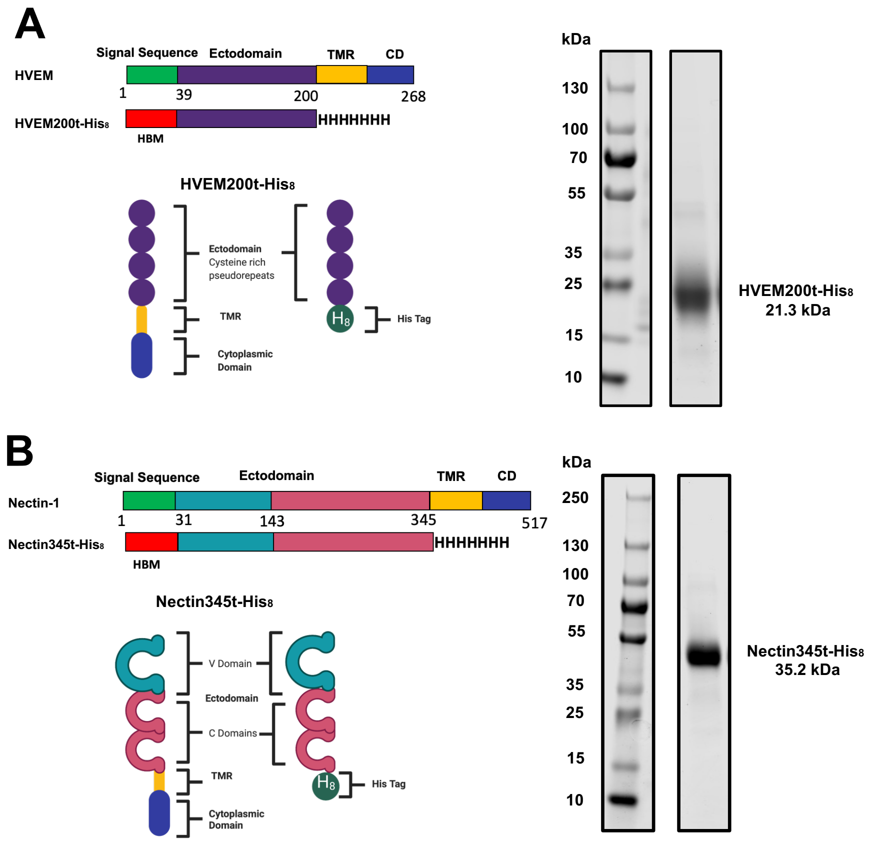

Supplementary Figure S3. HSV-1 receptors used in the bulk fusion assay. (A and B, left) Soluble HVEM **(A)** and nectin-1 **(B)** receptor constructs were used for fusion assays. Native signal peptide sequences were replaced with those of honeybee melittin. Transmembrane (TMR) and cytoplasmic domains (CD) were replaced with 8 histidines. (**A** and **B**, right) Coomassie gels show purified HVEM220t-His_8 **(A)** and Nectin345t-His_8 **(B)** used for fusion assays.

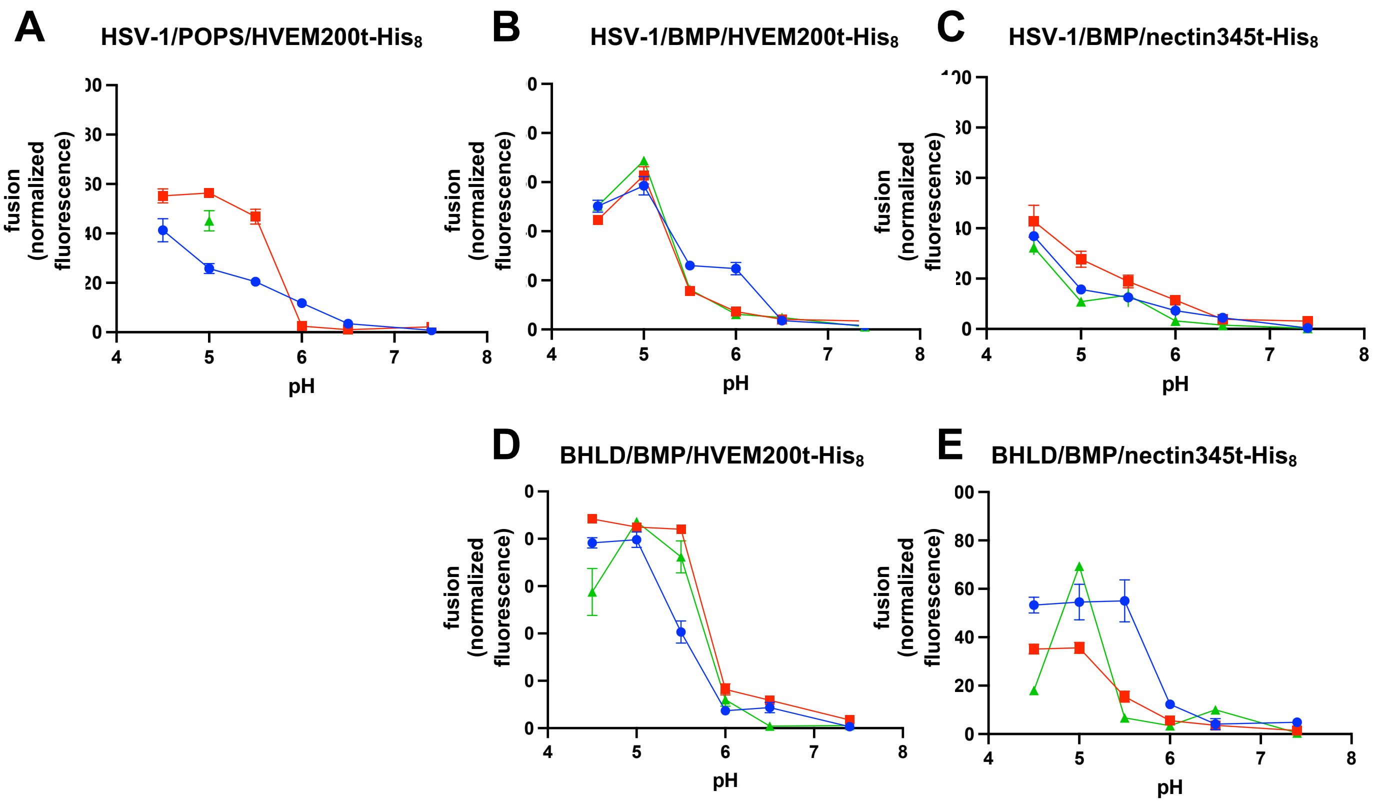

Supplementary Figure S4. Technical replicate variability of pH series fusion of HSV-1 and BHLD with HVEM200t-His$_8$ and Nectin 345t-His8. (A-C) HSV-1 and **(D and E)** BHLD total content mixing at 5 minutes post triggering (*t*= 5 min) with POPS (**A**) or BMP (**B-E**) liposomes (33% cholesterol, 33% POPC, 16.7% POPE, and 16.7% POPS/BMP) at different pH values in the presence of HVEM200t-His$_8$ or nectin345t-His$_8$. Shown are different biological replicates in red (square), blue (circles), or green (triangles), with each data point representing a mean value and error bars showing SEM.

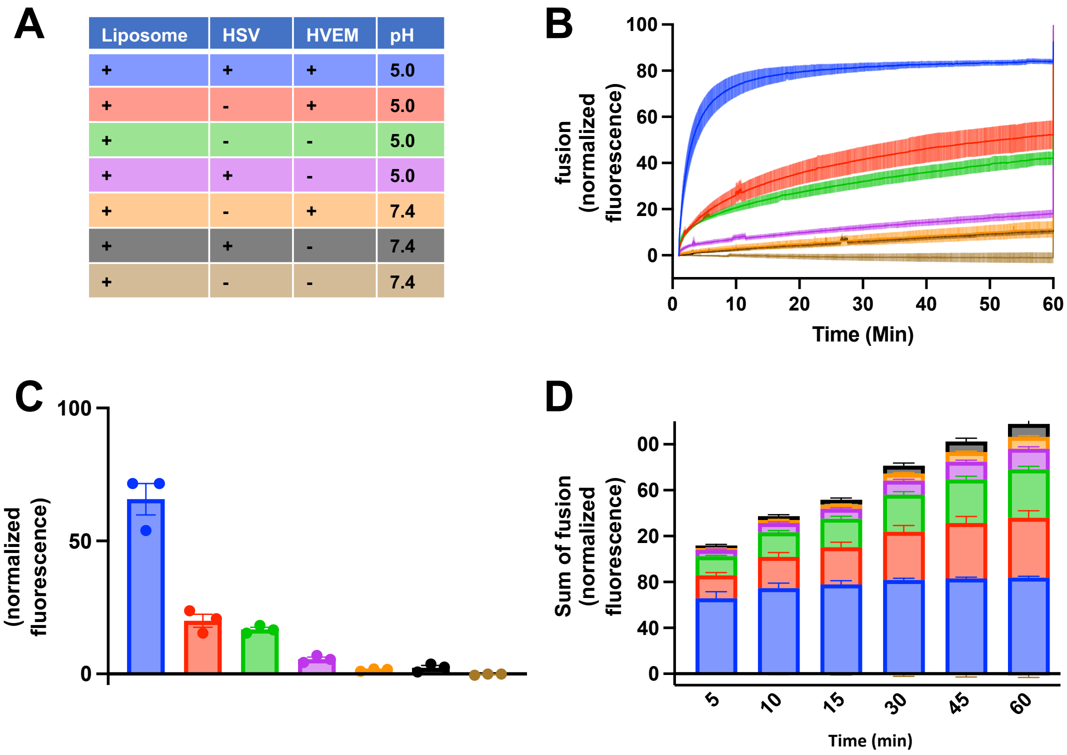

Liposome	HSV	HVEM	pH
+	+	+	5.0
+	-	+	5.0
+	-	-	5.0
+	+	-	5.0
+	-	+	7.4
+	+	-	7.4
+	-	-	7.4

Supplementary Figure S5. SRB-containing BMP LUVs are subject to leakage at later time points. (A) Table of the test conditions used for fusion experiments with color legend. (B) HSV-1, SRB-containing BMP LUVs, and HVEM200t-His$_8$ were incubated in different combinations for 1 min, at which point the reaction was triggered by a pH shift. After 60 min, the reaction was fully dequenched using 0.1% Triton X-100. Increase in SRB fluorescence due to its dilution and dequenching reported on fusion. Fluorescence was recorded and normalized to fully dequenched fluorescence by using the equation: $F_{normalized}=F_t\text{-}F_i\ /\ (F_{max}\text{-}F_i)^{-1}\ x\ 100\%$, where F_t is fluorescence at time *t*, F_i is initial fluorescence and F_{max} is fluorescence after the addition of Triton X-100. For each condition, representative traces of single biological replicates, each consisting of three technical replicates, are shown. Curves represent the mean values, and the shaded area, the SEM. **(C)** Extent of fusion at 5 min post-triggering (acidification). Each data point is a biological replicate representing a mean normalized fluorescence from 3 technical replicates. Bars represent the mean values, and the error bars, the SEM. Each condition was done in triplicate. *: $p < 0.05$, **: $p < 0.01$, ***: $p<0.001$ ****: $p<0.0001$. **(D)** Data in **(C)** are shown in a stacked format to compare relative fluorescence at specific time points.

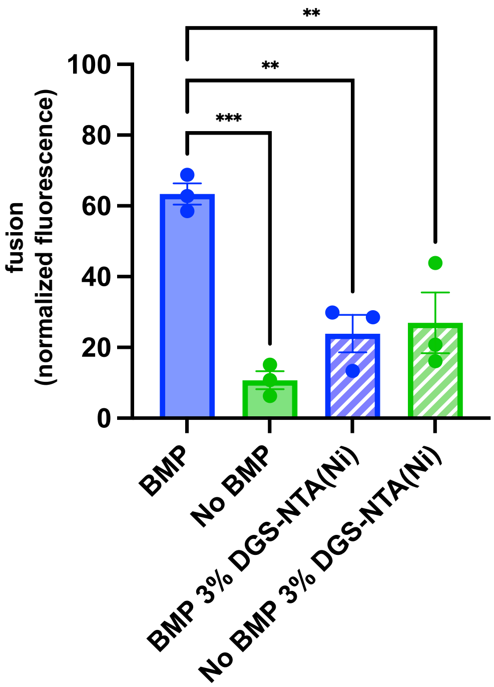

Supplementary Figure S6. Soluble receptors tethered to Ni-containing liposomes by histidine tags support fusion at low levels. HSV-1 was incubated with DGS-Ni-NTA containing BMP/No BMP liposomes and HVEM for 10 minutes prior to data collection at 37 °C. Baseline fluorescence was recorded for 1 min, at which point the reaction was triggered by a pH shift to 5.0. After 5 min, the reaction was fully dequenched at 0.1% Triton X-100. Fluorescence was recorded at 5 min post-triggering (acidification) and normalized to fully dequenched fluorescence by using the equation: $F_{normalized}=F_t-F_i\ /\ (F_{max}-F_i)^{-1}\ x\ 100\%$, where F_t is fluorescence at time t, F_i is initial fluorescence and F_{max} is fluorescence after the addition of Triton X-100. Each data point is a biological replicate representing a mean normalized dequenching value from three technical replicates. Bars represent the mean values and the error bars, the SEM.*: p < 0.05, **: p < 0.01, ***: p<0.001 ****: p<0.0001. BMP and No BMP data are the same as shown in Figure 4.

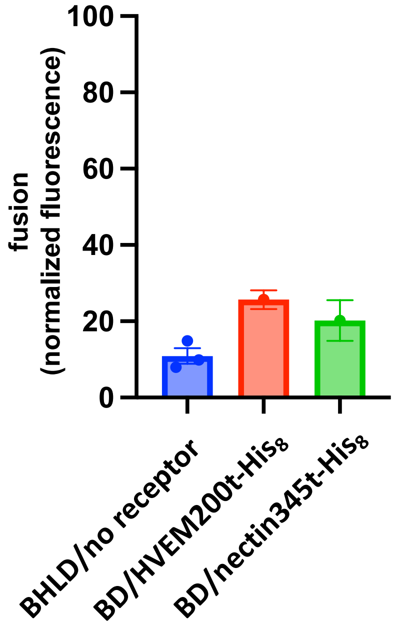

Supplementary Figure S7. VSVΔG-BHLD pseudotypes require receptor and the gH/gL complex for fusion. VSVΔG-BHLD was tested for fusion with SRB-containing BMP LUVs at pH 5.0 in the absence of any receptor. VSVΔG-BD, which lacks both gH and gL, was tested for fusion at pH 5.0 in the presence of HVEM200t-His$_8$ or nectin345t-His$_8$. Increase in SRB fluorescence due to its dilution and dequenching reported on fusion. Lipid composition is listed in Table 1. Fluorescence was recorded and normalized to fully de-quenched fluorescence by using the equation: $F_{normalized}=F_t-F_i$ / $(F_{max}-F_i)^{-1}$ x 100%, where F_t is fluorescence at time t, F_i is initial fluorescence and F_{max} is fluorescence after the addition of Triton X-100. Bars represent the mean values, data points individual technical replicates, and the error bars, the SEM. Each condition was done in biological (no receptor) or technical (ΔgHgL) triplicate.

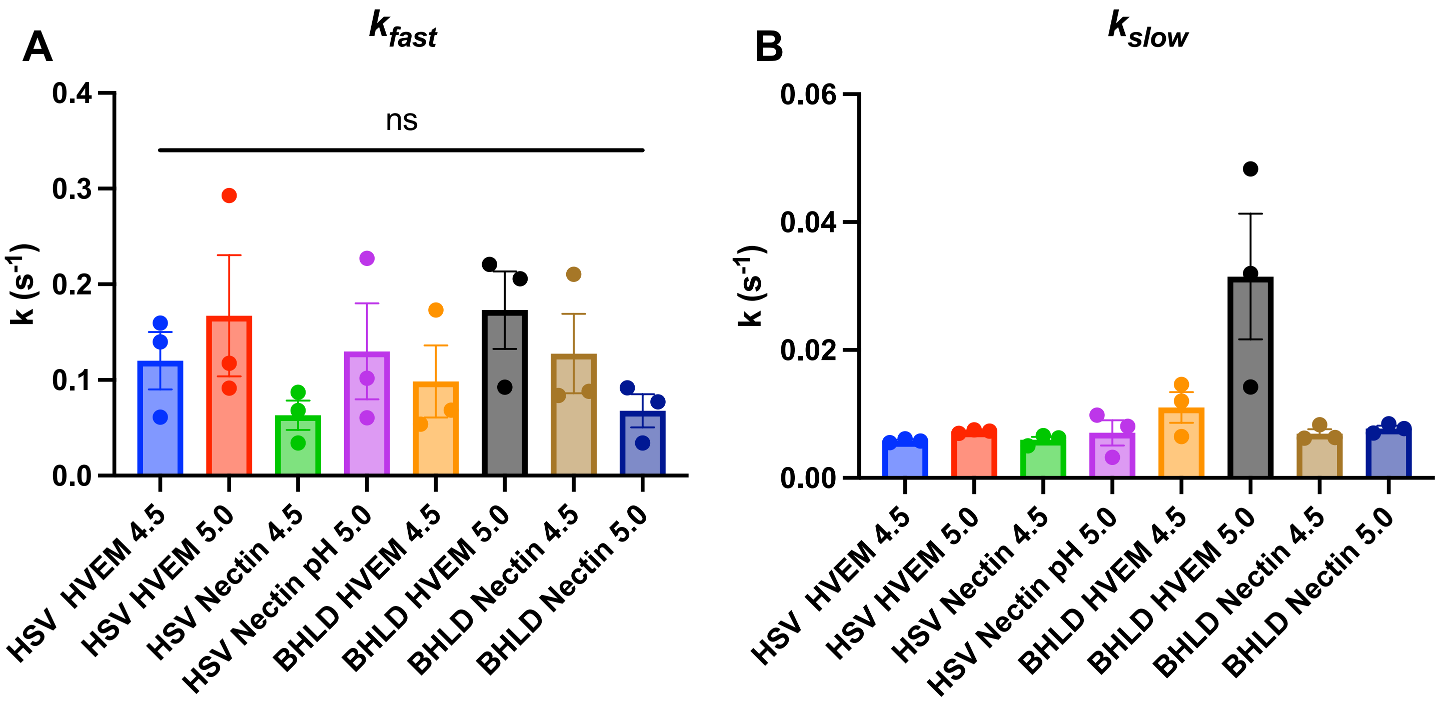

Supplementary Figure S8. Rate constants of SRB dequenching. Rate constants were determined by fitting the normalized data using the following two-phase (exponential) association analysis built into GraphPad PRISM9, $Y = Y_0 + \text{SpanFast} \times (1\text{-}e^{(-K_{Fast} * X)}) + \text{SpanSlow} \times (1\text{-}e^{(-K_{Slow} * X)})$, where $\text{SpanFast} = (\text{Plateau} - Y_0) \times \text{PercentFast} \times 0.01$ and $\text{SpanSlow} = (\text{Plateau} - Y_0) \times (100\text{-PercentFast}) \times 0.01$. Data points are fit values determined using the average biological trace, bar height is average value and error bars SEM.

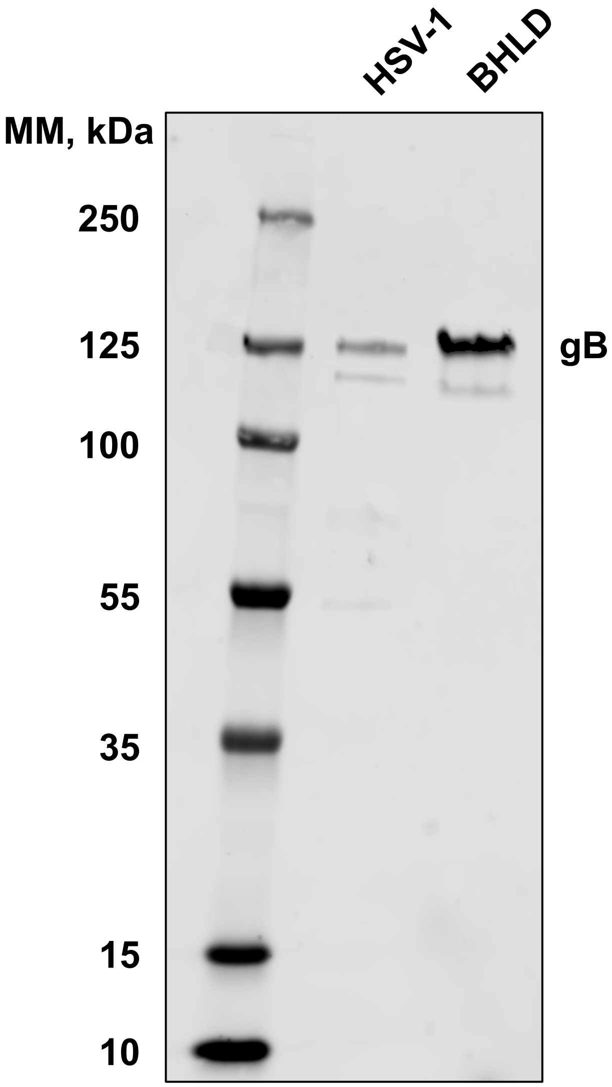

Supplementary Figure S9. BHLD particles contain more gB per particle compared to HSV-1. Polyclonal antibody R68 against HSV-1 gB was used with Licor IR800 secondary to determine levels of gB on a per-particle basis. Equivalent amounts of viral particles (~3 x10^7) were loaded using concentrations determined by light scattering.
